# Amick Again

**Published:** 1892-11

**Authors:** 


### EDITORIAL DEPARTMENT.

F. E. DANIEL, M. D., Editor.
A. J. SMITH. M. D., Galveston, Associate Editor.

### AMICK AGAIN.

When "A Chemical Cure for Consumption; by W. R. Amick, A. M., M. D., Professor of Ophthalmology in the Cincinnati College of Physicians and Surgeons, and formerly "Professor" of ever-so-many other things, made its appearance as an original article in the *Lancet-Clinic*, March 5, 1892, DANIEL'S TEXAS MEDICAL JOURNAL* was the very first to denounce it as a piece of clap-trap, and its author as a quack; and to score the *Lancet-Clinic* for permitting such flagrant disregard of all ethical principle and of professional decency to desecrate its pages. Other journals took it up, and the *Lancet-Clinic* hastened to publish a disclaimer; and stated in effect that the writer of the article had abused the confidence of that journal, and had gotten the article into its columns surreptitiously, as it were; that when he handed in the article, the editor, having every confidence in Dr. Amick's integrity, and soundness on the code question, sent it to the printers without examination, and that out of courtesy, as well as a matter of convenience—Amick's office being next to the printing office—Amick was permitted to read the proof. In that way, said the *Lancet-Clinic*, the article got into its pages without a knowledge of its real nature, on the part of the editor. The *Lancet-Clinic* later apologized to its many readers, and it was said that Amick was promptly relieved of his position in the college,—"kicked out of the profession," says the St. Louis *Weekly Review*.

Amick must be endowed with a "cheek of adamant." This article, some sixteen double-column pages, now appears, with its long array of almanac-like testimonials, as an advertisement in the *Maryland Medical Journal*, in the shape of a *reprint of an original article* from the *Lancet-Clinic*, of March 5, 1892 (as it really is; but, under the circumstances, it having been repudi-

*See Vol. vii, No. 10, Apl. 1892, page 333.

ated, its publication as such, is an outrage on the *L.-C.*, and on professional decency).

The *St. Louis Weekly Review* says that this advertisement was offered to a number of journals; or rather, that somebody wrote to a number of journals asking them to bid on a very large—an unprecedentedly large adv. contract, without stating what the matter was, and that very few journals bit at the tempting bait, the *Maryland Medical Journal* being one of the few, and the only one mentioned by the *Review*.

We feel slighted! They didn't invite the "Red Back" to put in a bid; they thought, perhaps, that we had already given the subject sufficient ventillation, and that they had better skip us,—and they did.

That so respectable a journal as the *Maryland Medical* should accept such an advertisement *at any price* is indeed surprising—and much to be regretted by those who have had as high opinion of the representative of the State of Patriotism and Chivalry—"Maryland my Maryland," as had the Red "Star of Texas." It only shows,—well, we'll be charitable and will not say what it shows.

Has the *Lancet-Clinic* no recourse at law to stop said proceedings? It is *libelous*. If nothing else will touch a sensitive spot in Amick's skin, which appears to be as thick as that of the Rhinoceros, the *L.-C.* might send for Culbertson to come back, the fighting editor, and let him try the application of a little rawhide.

